# Danger of Eating Too Little

**Published:** 1874-12

**Authors:** 


Danger of Eating Too Little. — Dr. Max von Pettenkofer disputes the rule laid down by so many physiologists, that so long as the body is able to perform its functions, even though suffering from hunger, to take more food is superfluous. Bichoff and Voitt fully demonstrated, by their experiments on nutrition, that the result of a nourishment so restricted is an actual state of want, a continual famine, a condition incompatible, in the long run, with the normal conditions of life. The body, says Pettenkofer, has need of a certain well being—of a small excess of nourishment — in order to preserve its strength and vigor, and what just prevents death from hunger is not sufficient.

